# Life-threatening *Mycobacterium intracellulare* pleuritis in an immunocompetent host: Case reports

**DOI:** 10.1097/MD.0000000000028342

**Published:** 2021-12-23

**Authors:** Bo-Gun Kho, Young-Ok Na, Hwa Kyung Park, Jae-Kyeong Lee, Hyung-Joo Oh, Ha-Young Park, Tae-Ok Kim, Hong-Joon Shin, Yong-Soo Kwon, Yu-Il Kim, Sung-Chul Lim

**Affiliations:** a Department of Internal Medicine, Chonnam National University Hospital, Gwangju, Republic of Korea; b Chonnam National University Medical School, Gwangju, Republic of Korea.

**Keywords:** bronchopleural fistula, *Mycobacterium intracellulare*, pleuritis

## Abstract

**Rationale:**

Nontuberculous mycobacteria (NTM)–associated pleuritis is a very rare disease. Here, we describe 2 cases of life-threatening *Mycobacterium intracellulare-*associated pleuritis in immunocompetent hosts.

**Patient concerns:**

A 78-year-old man with sudden onset-onset dyspnea (case 1) and an 80-year-old man with cough, sputum and fever (case 2) presented to our emergency room.

**Diagnoses:**

Both the patients were diagnosed with *Mycobacterium intracellulare*-associated pleuritis.

**Intervention:**

In case 1, the patient underwent intubation with mechanical ventilation due to hypoxemic respiratory failure. Daily azithromycin, rifampin and ethambutol, and intravenous amikacin 3 times a week was administered. In case 2, the patient received daily azithromycin, rifampin and ethambutol, and intravenous amikacin 3 times a week.

**Outcomes:**

In case 1, after receiving NTM treatment for 14 months, NTM-associated pleuritis was cured, with radiologic improvement. In case 2, however, bronchopleural fistula was developed. Despite tube drainage, air leak continued. The patient refused surgical management and eventually died of respiratory failure.

**Lessons:**

Pleural effusion arising from NTM lung disease located in the subpleural area should be considered a possible cause of NTM-associated pleuritis. Drainage and a multidrug regimen are required to treat NTM, and surgical treatment should be considered when complications occur.

## 1 Introduction

Nontuberculous mycobacteria (NTM) are a group of mycobacterium species other than *Mycobacterium* tuberculosis complex, *Mycobacterium* leprae and *Mycobacterium* ulcerans.^[1]^ The most common spectrum of NTM disease is pulmonary disease accounting for about 90% of all NTM disease.^[1]^ The incidence of NTM lung disease is increasing worldwide.^[2–4]^ However, NTM-associated pleuritis remains very rare.^[5–8]^ Here, we describe 2 cases of life-threatening *Mycobacterium intracellulare*–associated pleuritis in immunocompetent hosts.

## 2 Methods

This study was approved by the Institutional Review Board of the Chonnam National University Hospital (the number of approval: CNUH-EXP-2021-421). Written informed consent was obtained from each patient for publication of this case report and any accompanying images.

## 3 Case presentations

### 3.1 Case 1

**Figure 1:**
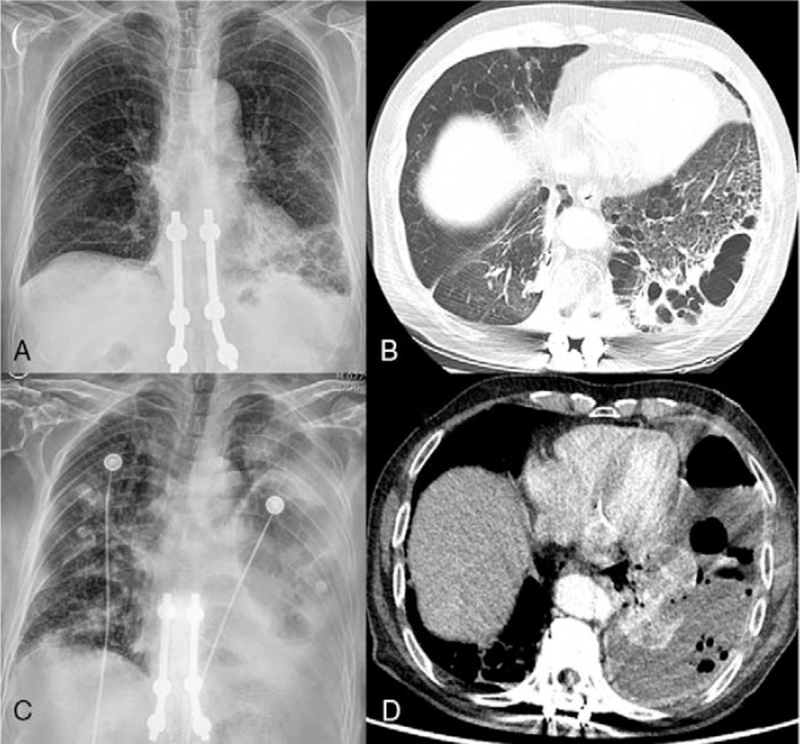
Chest radiograph and computed tomography (CT) images of case 1. (A) and (B) Images obtained 4 months before presentation. (C) and (D) Images obtained at presentation to the emergency room. (A) Chest radiograph showing consolidations in the left lower lobe. (B) Chest CT image showing a subpleural cavitary lesion with perilesional consolidation in the left lower lobe. (C) Chest radiograph showing a pleural effusion with air-fluid levels in the left hemithorax and consolidations in the right lung. (D) Chest CT images revealing a large pleural effusion with smooth wall thickening and enhancement suggestive of pleural empyema in the left lung.

A 78-year-old man presented to our emergency room with sudden-onset dyspnea. He was an ex-smoker with a 60 pack-year history. He also had a history of diabetes mellitus managed by medication and underwent spinal fusion surgery 10 years prior. He had recently undergone a bronchoscopic procedure to evaluate a cavitary lesion in the left lower lobe at a local clinic (Fig. 1A, B). He had taken antituberculous drugs empirically 7 days before presenting at the emergency room at the local clinic. He presented with a 1-day history of acute-onset fever, chills, purulent sputum, pleuritic chest pain, and dyspnea.

A physical examination revealed tachycardia at 120 beats/min. His blood pressure was 120/80 mm Hg, body temperature was 36.0°C, and respiratory rate was 28 breaths/min. A chest examination showed coarse crackles on the left lung.

The white blood cell (WBC) count was 9900/μL, with a neutrophil differential of 92.4%. The C-reactive protein level increased to 30.1 mg/dL (reference range, 0–0.6 mg/dL). Chest radiography and computed tomography revealed aggravating pneumonic consolidation and a cavity in the left lower lobe with a newly developed massive pleural effusion (Fig. 1C, D). On an arterial blood gas analysis, the partial pressure of oxygen was 97 mm Hg and partial pressure of carbon dioxide was 26 mm Hg through a face mask with 15 L/min of oxygen. The patient received tazobactam/piperacillin and underwent immediate pig-tail catheter insertion. A pleural fluid analysis revealed an exudate with a WBC count of 227700/mm^3^, neutrophil differential of 88%, lymphocyte differential of 10%, adenosine deaminase level of 179 IU/L, and pH of 5.0. The patient was admitted to the respiratory ward and continued treatment with antibiotics and drainage. However, on day 2, the patient was transferred to the intensive care unit and underwent intubation with mechanical ventilation due to acute hypoxemic respiratory failure. We decided to insert a chest tube instead of a pig-tail catheter and change the antibiotics to meropenem and vancomycin.

**Figure 2:**
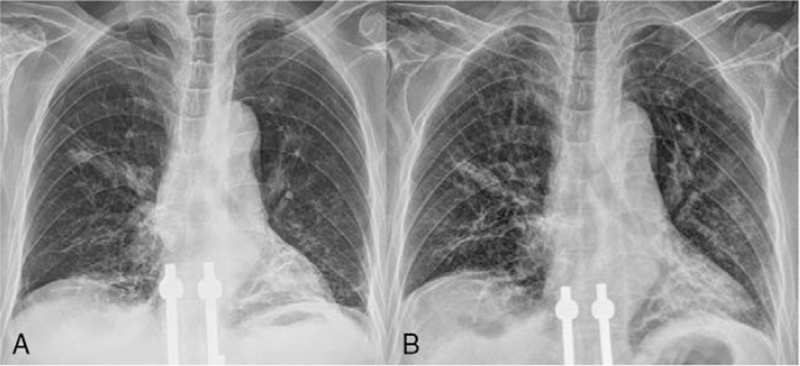
Chest radiographs taken (A) at the end of nontuberculous mycobacteria treatment; and (B) 1 year after the end of treatment.

On day 7, *Mycobacterium intracellulare* was identified at a local clinic in a specimen obtained via bronchial washing. The multidrug regimen consisting of daily azithromycin 250 mg, rifampin 600 mg, and ethambutol 800 mg and intravenous amikacin 1.0 g 3 times a week was administered to treat the *M. intracellulare* lung disease. A pleural fluid culture also revealed *M. intracellulare*. We conclude that the present empyema developed after rupture of the previous cavitary lung lesion associated with the *M. intracellulare* lung disease. We continued the anti-NTM treatment. Negative sputum culture conversion was achieved on day 27 of NTM treatment. On day 42, we removed the chest tube. On day 58, the patient was discharged home on long-term oxygen therapy. After 5 months of NTM treatment, azithromycin, rifampin, and ethambutol daily therapy were maintained without amikacin injections. The NTM treatment was finished after a total of 14 months (Fig. 2A). There was no evidence of NTM recurrence at 1 year after treatment completion (Fig. 2B).

### 3.2 Case 2

**Figure 3:**
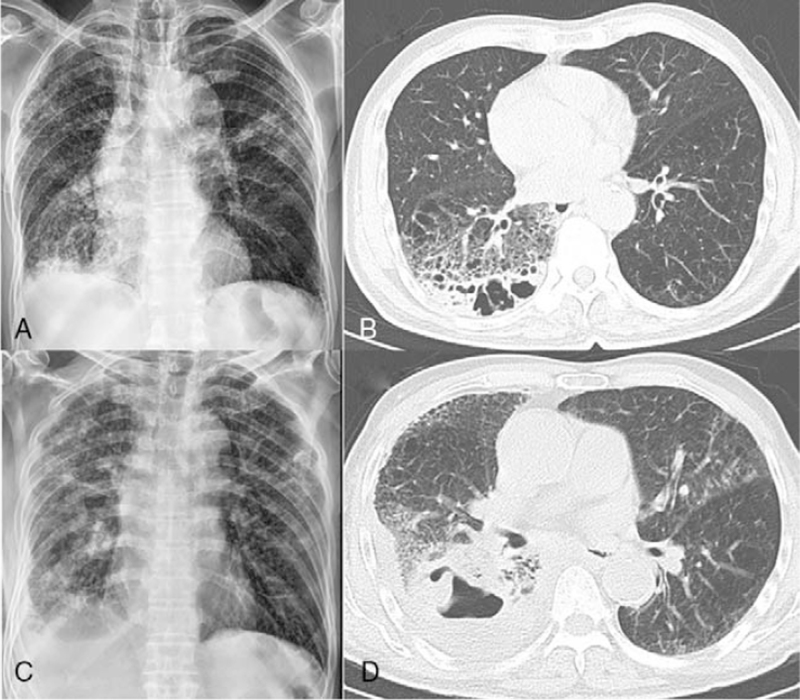
Chest radiographs and computed tomography (CT) images of case 2. (A) and (B) Images obtained 5 months before presentation. (C) and (D) Images obtained at presentation to the emergency room. (A) Chest radiograph showing consolidations in the right lower lobe. (B) Chest CT images showing a subpleural cavitary lesion with perilesional consolidation in the right lower lobe. (C) Chest radiograph showing a pleural effusion with consolidation in the right hemithorax. (D) Chest CT image revealing an increased subpleural cavitary lesion and pleural effusion in the right hemithorax.

An 80-year-old man presented to our emergency room with a 5-day history of cough, sputum, and fever. He was an ex-smoker with a 30 pack-years history. He also had a history of hypertension treated with medication. Five months prior, he was diagnosed with *M. intracellulare* lung disease with a subpleural cavitary lesion in the right lower lobe (Fig. 3A, B). However, he refused treatment for the NTM lung disease.

His blood pressure was 100/60 mm Hg and body temperature was 37.5°C. His pulse rate was 83 beats/min and respiratory rate was 20 breaths/min. A chest examination showed coarse crackles on the right lung.

The WBC count was 18500/μL, with a neutrophil differential of 80.6%. The C-reactive protein level was increased to 11.0 mg/dL. Chest radiography and computed tomography revealed aggravating pneumonic consolidation and a cavity in the right lower lobe with a newly developed pleural effusion (Fig. 3C, D). On the arterial blood gas analysis, the partial pressure of oxygen was 69 mm Hg and partial pressure of carbon dioxide was 25 mm Hg on room air.

**Figure 4:**
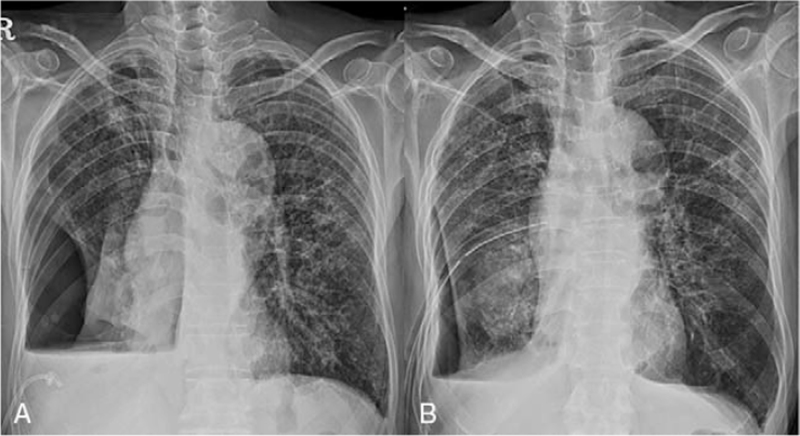
Chest radiographs showing (A) hydropneumothorax with pigtail catheter insertion in the right lung; and (B) hydropneumothorax with chest tube insertion in the right lung.

The patient received tazobactam/piperacillin and underwent immediate thoracentesis. A pleural fluid analysis revealed an exudate with a WBC count of 2807/mm^3^, neutrophil differential of 39%, lymphocyte differential of 40%, adenosine deaminase 69.6 of IU/L, and pH of 7.4. The patient was admitted to the respiratory ward and treatment with antibiotics and drainage was continued. The multidrug regimen consisting of daily azithromycin 250 mg, rifampin 600 mg, and ethambutol 800 mg and intravenous amikacin 1.0 g 3 times a week was administered to treat the *M. intracellulare* lung disease. A pleural fluid culture also revealed *M. intracellulare*. On day 11, a chest X-ray revealed spontaneous secondary pneumothorax at the right hemithorax (Fig. 4A). A pigtail catheter was inserted with chest wall suction. However, the pneumothorax did not improve; therefore, a chest tube was inserted on day 29 instead of the pigtail catheter. Negative sputum culture conversion was achieved after 6 weeks of NTM treatment. However, due to persistent air leaks, the right lung was not fully expanded, suggesting the presence of a complicated bronchopleural fistula (Fig. 4B). We recommended surgical management to the patient, but he refused. On day 90, he was transferred to a local clinic with chest tube. However, 28 days later, he died of acute respiratory failure.

## 4 Discussion

Here, we described 2 patients who developed life-threatening pleuritis complicated by *M. intracellulare* lung disease. In both cases, *M. intracellulare* lung disease with subpleural cavity preceded the development of pleuritis. Both cases achieved sputum culture conversion using the multidrug regimen; however, the patient in case 2 developed a complicated bronchopleural fistula and eventually died of respiratory failure.

The pleuritis associated with NTM is rare. Ando et al reported 15 cases (1.4%) of NTM pleuritis among 1044 patients with NTM lung disease.^[6]^ Park et al demonstrated 14 patients with NTM pleuritis in a 17-year retrospective study of the data of a single tertiary hospital.^[7]^ Naito et al and Yoon et al reported retrospective data of 12 patients over 15 years and 5 patients over 13 years in a single center each.^[5,8]^ The most common causative microorganism of NTM pleuritis is *M. intracellulare*.^[5,7–9]^ However, Ando et al reported that 9 of 15 patients with NTM pleuritis had *M. avium* infection.^[6]^ The infections in the 2 cases in this study were associated with *M. intracellulare*. The rate of treatment initiation due to the progression of NTM lung disease is higher in patients with *M. intracellulare* than in those with *M. avium*.^[10,11]^ Therefore, it is possible that *M. intracellulare* is a risk factor of the progression from NTM lung disease to pleuritis in cases of *M. avium* complex–associated lung disease. However, pleuritis associated with *Mycobacterium abscessus* complex, *Mycobacterium chelonae*, and *Mycobacterium fortuitum* has also been reported; therefore, further studies of the relationship between microorganisms and NTM pleuritis are required.^[12–14]^

Although the mechanism of NTM pleuritis is unclear, 2 theories have been proposed:^[15]^ invasion from NTM lung disease to the pleura; and perforation of the NTM lung disease resulting in leakage into the pleural space.^[6,10]^ In this study, both cases had subpleural cavities before developing NTM pleuritis. After development of the pleural effusion, bronchopleural fistula occurred in case 2. These findings support the latter hypothesis that the pleuritis arose from perforation of the existing NTM lung disease.^[6,8,10]^

The treatment regimens for *M. avium* complex*–*associated pleuritis have yet to be established. In this study, we treated the patients with a multidrug regimen consisting of azithromycin, rifampicin, and ethambutol and intravenous amikacin due to the advanced *M. avium* complex lung disease.^[16]^ Negative sputum culture conversion was achieved after 1 month (case 1) and 6 weeks (case 2) after the initiation of NTM treatment. Although the patient in case 1 required intubation with mechanical ventilation, the *M. intracellulare* diseases including lung disease and pleuritis were treated successfully without further complications. However, the patient in case 2 required a chest tube due to a complicated bronchopleural fistula. The patient in case 2 refused to undergo surgical treatment of the complicated bronchopleural fistula and eventually died of respiratory failure. Patients with NTM pleuritis often require surgical management. Naito et al reported that 7 of 12 patients with NTM-associated pleuritis required surgical management.^[5]^ Ando et al also reported that 6 of 15 patients with NTM-associated pleuritis required surgical treatment.^[6]^

## 5 Conclusion

Pleural effusion arising from NTM lung disease located in the subpleural area should be considered the possible cause of NTM-associated pleuritis. Drainage and multidrug regimen for NTM treatment are required, and surgical treatment should be considered in cases with complications.

## Author contributions

**Conceptualization:** Hong-Joon Shin.

**Data curation:** Bo-Gun Kho, Young-Ok Na, Hwa Kyung Park, Jae-Kyeong Lee, Hyung-Joo Oh.

**Formal analysis:** Ha-Young Park, Tae-Ok Kim, Yong-Soo Kwon, Yu-Il Kim, Sung-Chul Lim.

**Funding acquisition:** Hong-Joon Shin.

**Resources:** Young-Ok Na, Hwa Kyung Park, Jae-Kyeong Lee, Hyung-Joo Oh, Ha-Young Park, Tae-Ok Kim.

**Supervision:** Yong-Soo Kwon, Yu-Il Kim, Sung-Chul Lim.

**Validation:** Yong-Soo Kwon, Yu-Il Kim, Sung-Chul Lim.

**Writing – original draft:** Bo-Gun Kho.

**Writing – review & editing:** Bo-Gun Kho, Hong-Joon Shin.

## References

[1] Daley CL Iaccarino JM Lange C. Treatment of nontuberculous mycobacterial pulmonary disease: an official ATS/ERS/ESCMID/IDSA clinical practice guideline. Eur Respir J 2020;56:01–43. 10.1183/13993003.00535-2020 PMC8375621 32636299

[2] Hoefsloot W van Ingen J Andrejak C. The geographic diversity of nontuberculous mycobacteria isolated from pulmonary samples: an NTM-NET collaborative study. Eur Respir J 2013;42:1604–13. 23598956 10.1183/09031936.00149212

[3] Kendall BA Winthrop KL. Update on the epidemiology of pulmonary nontuberculous mycobacterial infections. Semin Respir Crit Care Med 2013;34:87–94. 23460008 10.1055/s-0033-1333567

[4] Kwon YS Koh WJ. Diagnosis of pulmonary tuberculosis and nontuberculous mycobacterial lung disease in Korea. Tuberc Respir Dis (Seoul) 2014;77:01–5. 10.4046/trd.2014.77.1.1 PMC4127406 25114696

[5] Naito M Maekura T Kurahara Y. Clinical features of nontuberculous mycobacterial pleurisy: a review of 12 cases. Intern Med 2018;57:13–6. 29033435 10.2169/internalmedicine.9119-17 PMC5799050

[6] Ando T Kawashima M Matsui H. Clinical features and prognosis of nontuberculous mycobacterial pleuritis. Respiration 2018;96:507–13. 30286448 10.1159/000490548 PMC6390458

[7] Park S Jo KW Lee SD Kim WS Shim TS. Clinical characteristics and treatment outcomes of pleural effusions in patients with nontuberculous mycobacterial disease. Respir Med 2017;133:36–41. 29173447 10.1016/j.rmed.2017.11.005

[8] Yoon HJ Chung MJ Lee KS Kim JS Park HY Koh WJ. Broncho-pleural fistula with hydropneumothorax at CT: diagnostic implications in mycobacterium avium complex lung disease with pleural involvement. Korean J Radiol 2016;17:295–301. 26957917 10.3348/kjr.2016.17.2.295 PMC4781771

[9] Wen P Wei M Xu YR Dong L. Clinical relevance and characteristics of nontuberculous mycobacterial pleuritis. Jpn J Infect Dis 2020;73:282–7. 32213718 10.7883/yoken.JJID.2019.314

[10] Moon SM Jhun BW Baek SY. Long-term natural history of non-cavitary nodular bronchiectatic nontuberculous mycobacterial pulmonary disease. Respir Med 2019;151:01–7. 10.1016/j.rmed.2019.03.014 31047103

[11] Koh WJ Jeong BH Jeon K. Clinical significance of the differentiation between Mycobacterium avium and Mycobacterium intracellulare in M avium complex lung disease. Chest 2012;142:1482–8. 22628488 10.1378/chest.12-0494

[12] Fabbian F De Giorgi A Pala M Fratti D Contini C. Pleural effusion in an immunocompetent woman caused by Mycobacterium fortuitum. J Med Microbiol 2011;60:1375–8. 21459911 10.1099/jmm.0.024737-0

[13] Hsieh HC Lu PL Chen TC Chang K Chen YH. Mycobacterium chelonae empyema in an immunocompetent patient. J Med Microbiol 2008;57:664–7. 18436603 10.1099/jmm.0.47574-0

[14] Fairhurst RM Kubak BM Shpiner RB Levine MS Pegues DA Ardehali A. Mycobacterium abscessus empyema in a lung transplant recipient. J Heart Lung Transplant 2002;21:391–4. 11897529 10.1016/s1053-2498(01)00339-4

[15] Kotani K Hirose Y Endo S Yamamoto H Makihara S. Surgical treatment of atypical Mycobacterium intracellulare infection with chronic empyema: a case report. J Thorac Cardiovasc Surg 2005;130:907–8. 16153958 10.1016/j.jtcvs.2005.02.054

[16] Griffith DE Aksamit T Brown-Elliott BA. An official ATS/IDSA statement: diagnosis, treatment, and prevention of nontuberculous mycobacterial diseases. Am J Respir Crit Care Med 2007;175:367–416. 17277290 10.1164/rccm.200604-571ST

